# *Batrachedra nuciferae*, an Inflorescence-Feeding Moth Associated with Coconut, *Cocos nucifera*, and Palmiste, *Roystonea oleracea*, in Trinidad, West Indies

**DOI:** 10.1673/031.013.12401

**Published:** 2013-11-05

**Authors:** Matthew J. W. Cock

**Affiliations:** CABI E-UK, Bakeham Lane, Egham, TW20 9TY, UK

**Keywords:** Arecaceae, Batrachedridae, caterpillar, Lepidoptera, palms, parasitoid, pollen

## Abstract

In 2006, *Batrachedra nuciferae* Hodges (Lepidoptera: Batrachedridae) was the first phytophagous insect to be reported from inflorescences of coconut, *Cocos nucifera* L. (Arecales: Arecaceae), in Trinidad, West Indies. At that time, it was suggested to be an introduced species contributing to decreasing coconut yields on the island and potentially a threat to other palms. In this preliminary study, inflorescences of coconut, seven indigenous palms, and six exotic ornamental palms were surveyed in several areas of Trinidad. Caterpillars of more than 10 species of Lepidoptera were found and reared through to the adult stage. *Batrachedra nuciferae* was positively identified. It was concluded that the caterpillars of *B. nuciferae* feed on pollen in the male flowers of coconut and palmiste or royal palm, *Roystonea oleracea* (Jacquin) O.F. Cook. There was no evidence that *B. nuciferae* bred on any of the other palms surveyed, but it is not conclusive that they do not do so. A parasitoid, *Apanteles* (*sensu lato*) sp. (Hymenoptera: Braconidae), of *B. nuciferae* was reared. On available information, *B. nuciferae* is more likely to be an indigenous species that has hitherto been overlooked than an introduced species. In view of what is known about damage-yield relationships and biological control agents, *B. nuciferae* is unlikely to cause yield losses to coconut, so control measures are not justified.

## Introduction

*Batrachedra nuciferae* Hodges (Lepidoptera: Batrachedridae) is a newly reported pest of coconut, *Cocos nucifera* L. (Arecales: Arecaceae), on the island of Trinidad apparently introduced at some time prior to 2006. Since *B. nuciferae* was already known as a pest of coconut and other palms in Brazil, concerns have been raised locally about its potential impact onHistory, distribution, and biology of *Bactrachedra nuciferae* coconuts and indigenous palms in Trinidad and Tobago, and the threat it may present in the wider Caribbean.

### History, distribution, and biology of *Bactrachedra nuciferae*

*Bactrachedra nuciferae*, which is referred to as the coconut moth in Trinidad and Tobago (MALMR 2008), was first recognised by Bondar (1940a, b), who described its biology in Bahia State, Brazil, under the incorrect name of *B. perobtusa* Meyrick. It was only in Hodges' (1966) revision of the American *Batrachedra* that the species was first described, based on Bondar's material reared from coconut. In 1998, *B. nuciferae* was recorded from coconut in Venezuela (Arnal et al. 1998), and in 2006 it was reported from Trinidad as a new pest of coconut (MALMR 2006, 2008).

Bondar (1940a, b) studied the insects associated with coconut inflorescence in Bahia, Brazil. He reported that the caterpillars of *B. nuciferae* rest in the male flowers of coconut, where they feed on pollen, and they are also common in the flowers of several other palms: *Syagrus coronata* (= *Cocos coronata*), *S. vagans* (= *C. vagans*), *S. schizophylla* (= *C. schizophylla*), *Attalea funifera*, and *A. piassabossu*. Bondar considered that the damage to male flowers reduced the probability of fertilization of female flowers and hence could adversely affect nut production, but presented no evidence for this conclusion. He gives brief descriptions of the caterpillar, pupa, and cocoon.

Since 1940, there was no published work on the coconut moth, apart from Hodges' (1966) description of *B. nuciferae*, until the work of S. Sanchéz-Soto this century. The moth was the subject of his research thesis (Sánchez Soto 2004) and publications on the distribution (Sánchez-Soto and Nakano 2002, 2004a), morphology (Sánchez-Soto and Nakano 2004b), and biology (Sánchez-Soto and Nakano 2008). The egg, caterpillar (including chaetotaxy), pupa, and adult are illustrated with line drawings in both Sánchez Soto (2004) and Sánchez-Soto and Nakano (2004b). Sánchez-Soto and Nakano (2008) reported that in captivity, development to adult takes 22 days, and there is an average pre-oviposition period of 2.6 days, followed by 11.3 days of oviposition during which an average of 31.5 eggs are laid.

### Damage caused by *Batrachedra nuciferae* to coconut

In contrast to Bondar's (1940a, 1940b) observations that the caterpillars feed only on coconut pollen, Arnal et al. (1998) reared *B. nuciferae* on male and female flowers and stated that it feeds on both. However, this was a brief description of a rearing method on inflorescences to confirm the presence of the moth in several parts of Venezuela, and it is not clear if careful observations were made. Carneiro et al. (2004) provided a summary of earlier observations and reported that during the dry season all male flowers may be destroyed. They state that in the Município de Parnaíba, Piauí, Northeast Brazil, the caterpillars feed on both male and female flowers and include a photograph of damaged female flowers, which certainly look in poor condition, although exactly what form the damage takes is not clear, whether general abrasion and fouling, surface feeding, or boring into the flower/nutlet. Although the observations of Carneiro et al. (2004) were based on reliably identified adult moths, no detailed observations seem to have been made, and the possibility of other insects being involved in causing the damage was not considered. Thus, in spite of these two reports, detailed observations of damage to female flowers positively linked to caterpillars of *B. nuciferae* are not available.

The pollination system of most palms, including coconut, is unknown or inadequately understood, and the impact of inflorescence feeding insects on fruit production is also poorly understood, even in the case of coconut, an important crop (Moore 2001). It is generally thought that damage to male flowers alone is unlikely to be significant to the extent of limiting fertilisation, especially where insects are implicated in the pollination mechanism. For pollen availability to limit fertilisation, it is likely that all or most male flowers would need to be prevented from producing pollen over large areas over an extended period, and this level of damage has not been reported.

Damage to female flowers could lead to direct losses of harvestable nuts. However, this is not well understood either (Taylor 1930; Corbett 1931; Cock et al 1987; Waterhouse and Norris 1987). Most female flowers are fertilised, but many abort naturally or are lost as early nutfall. There are mechanisms whereby coconut palms through nutfall can reduce the number of nuts brought to maturity in response to poor conditions, lack of nutrients, damage to the fronds, etc. In principle, this also means that there is scope for the palms to compensate for early damage to female flowers by insects.

### Objectives of this study

A preliminary survey of the incidence of Lepidoptera in the flowers of coconuts and other selected palms in Trinidad was carried out. This survey was to generate information to confirm the presence of *B. nuciferae* in Trinidad and to provide background to assess the threat to coconut and other palms and the need for prevention of spread and management of the moth.

## Materials and Methods

### Field collections

All field work was carried out in October 2011. Before making surveys, methods were tested on coconut palms in Curepe (a suburban area between Port of Spain and Arima), Centeno (mixed agricultural land south of Arima), and Waller Field (a mixed plantation east of Arima), which produced material for identification and confirmed the biology and damage symptoms of *B. nuciferae* on coconut. Three sites were prioritised for survey in Trinidad: Nariva Swamp, Aripo Savannah, and the Royal Botanic Gardens, Port of Spain. Nariva Swamp is an important biodiversity area in Trinidad. Samples were taken in the Bush Bush Island nature reserve and at Kernahan, a small community in the southern part of the swamp. Aripo Savannah is also a protected area, notable for its unusual savannah-like vegetation. The indigenous moriche palm *Mauritia flexuosa* L.f. is an important component of the flora. The Royal Botanic Gardens grow a selection of indigenous and introduced palms.

At each site, the procedure was as follows. A search was made on foot or from a vehicle to locate flowering palms in the survey area. The palms were identified from Comeau et al. (2003) and local knowledge. The stage of the inflorescence (pollen release or soon after being preferred) and its accessibility by ladder or climbing was assessed. Where no material to sample could be located, some old inflorescences were selected, as they would show indications of any damage by Lepidoptera. Suitable palms were then sampled, either from the ground for short palms, using a 5 m ladder for some, and a bamboo ladder and climber for *M. flexuosa* at Aripo Savannah. Each sample was a whole inflorescence and was placed in prepared sample bags (cotton cloth bags, measuring 112 cm deep × 77 cm wide, with a drawstring closure). Where possible, the inflorescence was cut from the palm with minimum vibration (which might cause male flowers to drop) using secateurs or long handled clippers, and the detached inflorescence was placed directly into a sample bag. This process was not always practical due to the difficulty of access or the toughness of the rachis, so in some cases a sample bag was placed over the whole inflorescence before cutting the stem with a machete. In the case of the samples from *M. flexuosa* and *Sabal mauritiiformis* (Karsten) Grisebach and Wendland, the inflorescences were too big for the sample bags and so they were cut and lowered to the ground as gently as possible, and then cut up and placed in the sample bag. In one case, a *Euterpe precatoria* Martius was felled to obtain a sample. The samples were labelled and placed in a chest cool box for transport back to the laboratory. When samples exceeded the capacity of the cool box, they were kept in the shade and then taken to the laboratory in an air-conditioned vehicle. At each site, pictures were taken of the palm, its inflorescence and other diagnostic features, and any other features considered worth recording. Where there was any doubt about the identity of the palm, specimens of the leaves, inflorescence, and fruit were taken to facilitate later identification. A GPS was used to record the position, elevation, and time of each sample. The palms and sample inflorescences obtained are given in Table 1.

**Table 1.**
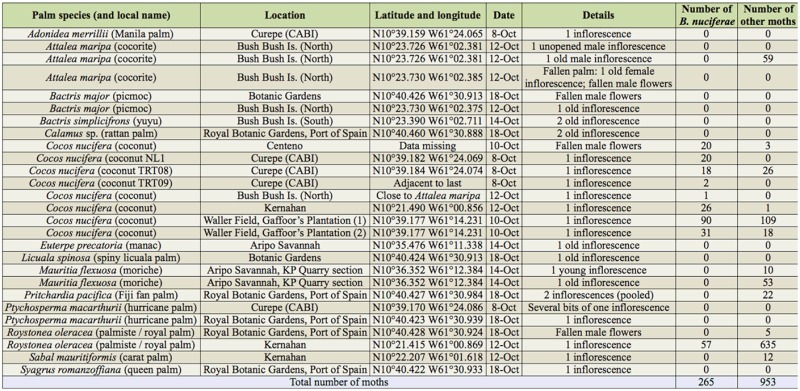
Collections of palm inflorescences made in Trinidad, 8–18 October 2011, and the adult *Bactrachedra nuciferae* and other moths reared from them until 22 December 2011. All sites are in lowland areas, at altitudes of less than 50m.

### Laboratory methods

On return to the laboratory, the sample bags containing inflorescence samples were placed in an air-conditioned room overnight and processed the next day. For each sample, the following steps were followed. The stage of flowering was recorded, and a photograph was taken of the entire inflorescence and any parts that were considered of interest, e.g., details of inflorescence and damage symptoms. Part or all of the inflorescence was examined by eye and under a binocular microscope to look for symptoms of Lepidoptera (presence of caterpillars or pupae, silk shelters or cocoons, feeding damage, frass, or droppings). Samples were taken of any living caterpillars or cocoons and either placed in individual vials (30–45 mL) or in groups in containers (14 diameter × 8.5 cm height round containers or 16.5 × 12.5 × 7.5 cm height rectangular boxes). Notes were made on what was found, and observations were quantified as practical, e.g., results per primary, secondary, or tertiary branch, number per 10 flowers, etc.

The remains of the inflorescence were placed in an emergence box and monitored for emergence of moths over more than two months. An emergence box is a cardboard box (up to 57 × 40 × 64 cm in size) with the seams sealed with masking tape to exclude ants and prevent escape of insects. Two round holes are cut in one side exactly large enough to fit a clear plastic or glass vial, which is then inserted with the open end just fitting into the hole and the closed end projecting. The box is placed in a room lit with fluorescent lighting and kept at ambient temperatures. Moths and most other insects are attracted to light, and so insects in the inflorescence or insects that complete their development there are attracted into the transparent vials from where they can be collected. The emergence boxes were checked several times a day, and when moths were found in the transparent vials, the vial was carefully removed, capped, and labelled with the collection and date of emergence. A record sheet on each box was used to record the dates of emergence and provisional division of the moths into *B. nuciferae* and other species. On 22 December, only a very small number of moths had emerged in the previous week from two of the emergence boxes, so the boxes were discarded.

The subsamples of living material from the first examination of the inflorescence were either placed in individual vials or in ventilated plastic boxes. The former did not prove satisfactory, perhaps because ventilation was inadequate, and most caterpillars thus treated did not complete development. The ventilated plastic boxes were more successful, and moths were successfully reared in these.

The reared moths were placed in a freezer (approximately -18° C) at least overnight before pinning, spreading, and staging using normal techniques for very small moths (e.g., Tagestad 1974). A selection was dissected using standard techniques (e.g., Robinson 1976) and used for identification by comparison with Hodges (1966).

**Figure 1.**
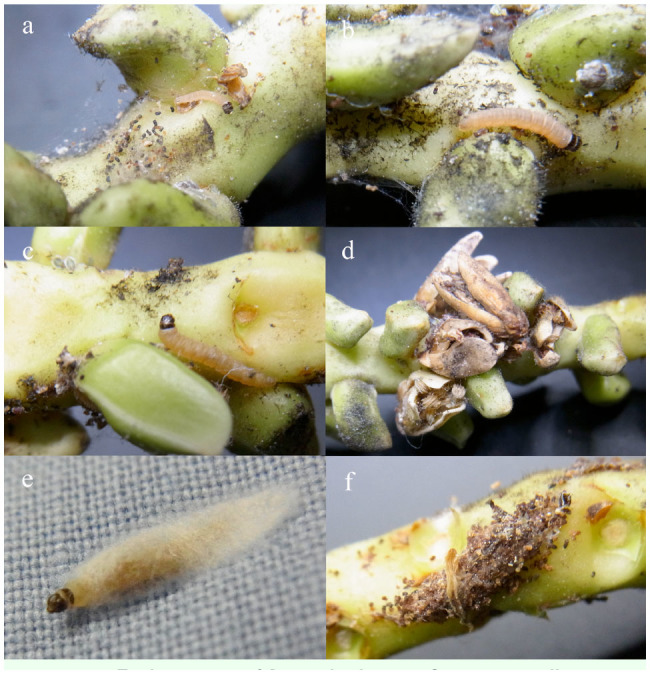
Early stages of *Batrachedra nuciferae*. a: small caterpillar at base of male flower, with frass speckled webbing to the left; b: larger caterpillar with trace of webbing to left of lower male flower; c: mature caterpillar, webbing, and frass bottom left; d: dead male flowers tied to inflorescence with silk; e: caterpillar spinning cocoon on surface of cloth field collection bag; f: typical cocoon formed on inflorescence branch, speckled with frass and small pieces of debris. High quality figures are available online.

## Results

In total, 26 palms were surveyed, including 8 *Cocos nucifera* (Table 1). From these, 265 *B. nuciferae* and 953 other moths were reared (Table 1).

The caterpillars, feeding style, and cocoons of *B. nuciferae* were found to be distinctive, enabling the early stages and damage of this species to be separated from other Lepidoptera found in the palm inflorescences. Caterpillars were characterised as white to pink, with the head and dorsal plate on the prothorax dark (Figure 1a–c, e), as described by Sánchez Soto (2004) and Sánchez-Soto and Nakano (2004b). The caterpillars construct shelter-tunnels with silk webbing among the male coconut flowers, usually incorporating scattered frass pellets (Figure 1a–c). As male flowers dehisce, some are attached by the silk webbing and so remain attached to the inflorescence branch as they turn brown and dry (Figure 1d). Caterpillars were found inside male flowers that had started to open and remained within the flowers until the contents were consumed. No evidence was seen that these caterpillars made holes in the male flowers or damaged female flowers, although more observations, especially at high caterpillar densities, would be desirable to confirm this.

Pupation was in a double, white silk cocoon, either on a solid substrate such as the inflorescence branch or among fallen male flowers. When infested male flowers were held in a plastic container, the cocoon was normally formed on the base of the container, and in the case of a transparent container, was readily visible through the container. The surface of the cocoon was normally covered with scattered frass pellets or small fragments of flowers attached to the silk, making it distinctive (Figure 1f). Some that were formed in the collection bags overnight on returning from the field were formed without attachments (Figure 1e).

**Figure 2.**
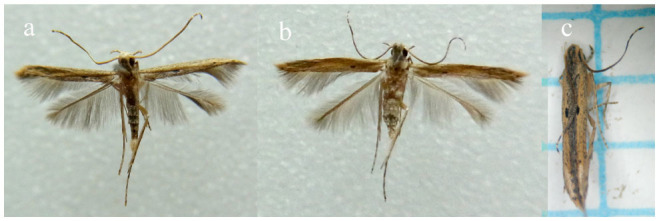
Adult *Batrachedra nuciferae*: a: male (wingspan 8.3 mm); b: female (wingspan 10.2 mm); c: dead adult with wings held parallel to body, giving an impression of the moth in life. (Photo credit: Dwayne H. Burris). High quality figures are available online.

**Figure 3.**
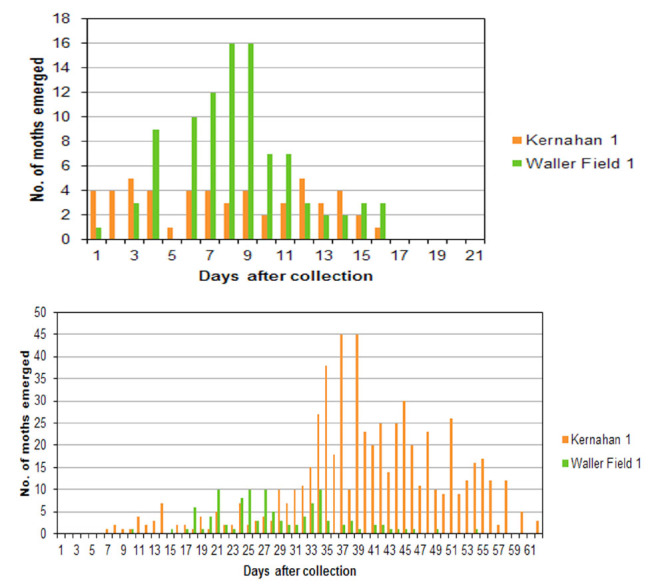
Emergence of *Batrachedra nuciferae* (above) and other moths (below) from emergence boxes. Kernahan 1: *Roystonea oleracea* (palmiste), collected 12 October 2011; Waller Field: *Cocos nucifera* (coconut) sample 1, collected 10 October 2011. High quality figures are available online.

The adult moths were as characterised by Sánchez Soto (2004) and Sánchez-Soto and Nakano (2004b), with very narrow, elongate, fringed wings (Figure 2). They rest with the head end raised above the substrate and the wing tips resting on the substrate (see Sánchez Soto 2004, Figure 7). The long narrow wings are held tightly against the body so that the moth appears long and thin (Figure 2c). The combination of colour, size, markings, and resting position enabled adult *B. nuciferae* to be distinguished from other Lepidoptera that emerged from the survey samples. Adult moths were thus provisionally identified as *B. nuciferae*. Pinned specimens were critically examined, and selected female specimens were dissected and compared with Hodges (1996, Figure 129) to confirm the identification. The size, shape, and surface structure of the signum were found to be diagnostic. This identification was confirmed by a USDA Lepidoptera taxonomist at the United States National Museum based on an examination of the male and female genitalia and comparison with reference material in the museum.

### Coconut

Adults of *B. nuciferae* were reared from coconut at all localities at which coconut was surveyed (Curepe, Centeno, Waller Field, Bush Bush, and Kernahan), but were not found at the Royal Botanic Gardens, where coconut could not be surveyed. The numbers obtained were variable (Table 1). Only 1 specimen was obtained from Bush Bush, but *B. nuciferae* was common at Curepe and Kernahan and very common at Waller Field (90 adults from inflorescence 1).

Most adult moths emerged within 15 days of collection of the inflorescence (Figure 3 top), and none took more than 23 days in any sample, whereas other species of moths continued to emerge for two months (Figure 3 bottom).

Ants and predatory wasps were common in the inflorescences sampled in the field. An *Apanteles* (*sensu lato*) sp. parasitic wasp (Hymenoptera: Braconidae) was reared from *B. nuciferae*, but was not identified. It is referred to as *Apanteles* (*sensu lato*) sp. because although the genus *Apanteles* has been divided into a large number of genera, it is still easily recognisable and familiar, whereas the new genera are not. The parasitoids attack the caterpillars before maturity, and the mature parasitoid larva only emerges once the caterpillar has spun its cocoon and developed to the prepupa stage. The parasitoid larva then spins a white cocoon within the *B. nuciferae* cocoon and emerges at about the same time as the adult moth would have. When cocoons were formed on a translucent rearing container, the parasitoid cocoons were readily visible through the container and host cocoon. *Apanteles* sp. were thus obtained from the Centeno coconut sample, direct from the *B. nuciferae* cocoons. Of 16 cocoons reared from this sample, 4 were parasitized.

### Palmiste

One inflorescence from a relatively short palmiste, *Roystonea oleracea* (Jacquin) O.F. Cook at Kernahan was included in the survey. Each inflorescence contains both male and female flowers, and an unusual feature of the genus is that the inflorescence is packed with millions of very small, fluffy, branched hairs before the spathe opens (Henderson et al. 1995). Insects such as bees are implicated in pollination of the congeneric *R. regia* (Zona 2000). In this inflorescence, the male flowers had recently dropped and the female flowers were still small. Many of the male flowers and the fluffy matrix were caught up in the inflorescence and the spathe, which was still in place below the inflorescence.

Examination of the male flowers and fluffy matrix revealed what appeared to be *Bactrachedra* caterpillars along with what were definitely *Batrachedra* sp. cocoons, as well as caterpillars of at least two other species. Adult *B. nuciferae* started emerging on the day of collection and continued to do so in the emergence box for two weeks, producing 57 adults. More than 600 adults of at least 5 other species of moths were reared from this collection over the next two months. Although large numbers of moths reared from this single inflorescence, most emerged after the *Batrachedra nuciferae* adults (Figure 3), so they were probably not feeding on pollen. It seems likely that the fluffy matrix contained in inflorescences of *R. oleracea* provides a food source that supports many caterpillars of some of these moths. A small sample of fallen male flowers of *R. oleracea* from the Royal Botanic Gardens produced just 5 adult moths of 2 species, but no *B. nuciferae*.

### Other palms

*Attalea maripa* (Aublet) Martius is common at Bush Bush, but only an unopened spathe, an old dead male inflorescence, and a long-dead female inflorescence from a fallen palm could be included in the survey. The contents of the unopened spathe were unblemished, with no signs of insect damage. The very old female inflorescence showed no recent sign of Lepidoptera damage and was discarded after examination in the laboratory on the basis that any Lepidoptera still associated with it would be saprophytic. The old male inflorescence, in which the flower parts, including the long pollen- bearing stamens (Henderson et al. 1995), were completely dead and dry, showing a great deal of old feeding damage by Lepidoptera based on the amount of frass and webbing that was observed. Careful search revealed no indication of *Batrachedra* type cocoons on the stems or among the flower remains, and no *Batrachedra* spp. moths were obtained by emergence box, although other moths were reared.

Most *Bactris major* Jacquin palms at Bush Bush Island were not fruiting or flowering on 12 October 2011, but one that had just flowered was sampled. In the laboratory, no trace of Lepidoptera feeding could be detected on this inflorescence, and nothing emerged subsequently from the emergence box. At the Royal Botanic Gardens, a stand of *B. major* showed fruiting, and inflorescences similar to those found at Bush Bush Island. An inflorescence from which the male flowers had recently been shed showed no signs of Lepidoptera feeding. Examination of fieldcollected, newly-dropped, male flowers in the laboratory showed that they seemed fresh and had pollen remaining, so they should have been suitable food for Lepidoptera such as *Batrachedra* spp. However, no sign of Lepidoptera feeding was found when 10 flowers were opened, and the remainder examined externally, and no moths subsequently emerged from this material.

At Bush Bush, no *Bactris simplicifrons* Martius could be found with open male flowers, although 2 were found that had recently dropped their male flowers. These were examined in the laboratory and no trace of Lepidoptera or other insect damage could be seen.

Examination of the branches on an inflorescence of *E. precatoria* from which the male flowers had dropped showed no signs of Lepidoptera damage and specifically no sign of male flowers attached to the inflorescence by silk, or the typical cocoons of *Batrachedra* spp. No moths were obtained from the emergence box.

At Aripo Savannah, 1 male moriche palm, *M. flexuosa*, was found that was short enough to sample. It had several old, dead, dark brown inflorescences and several young, yellowgreen ones that had not yet flowered. Both developmental stage inflorescences were included in the survey. The young inflorescence showed no sign of Lepidoptera damage when inspected, although small numbers of 3 species of moths other than *B. nuciferae* were obtained by emergence box. Some feeding damage and very small caterpillars were found under the bracts of the very old inflorescence, but there were no signs of *Batrachedra* spp. and none were reared from the emergence box.

*Sabal mauritiiformis* is unusual among the indigenous palms surveyed in that the small flowers are bisexual (Henderson et al. 1995). One inflorescence that included open male flowers was sampled adjacent to the Forestry Division facility at Kernahan. Caterpillars of an unidentified *Batrachedra* sp. were found and reared from this inflorescence, but no other Lepidoptera were found or reared.

The remaining palms are all introduced ornamentals in Trinidad: Manila palm, *Adonidea merrillii* (Beccari), rattan palm, *Calamus* sp., mangrove fan palm, *Licuala spinosa* Roxburgh**, Fiji fan palm, *Pritchardia pacifica*, hurricane palm, *Ptychosperma macarthurii* (H. Wendland ex H.J. Veitch) H. Wendland ex Hook.f., and queen palm, *Syagrus romanzoffiana* (Chamisso) Glassman.

Although *B. nuciferae* caterpillars were found on coconut inflorescences about 25 m away from the *A. merrillii* surveyed at Curepe, no sign of Lepidoptera feeding could be found in the inflorescence of *A. merrillii*, and none were obtained by emergence box.

The survey sample from *Calamus* sp. was 2 old, dry, male inflorescences, comprising a densely packed mass of dry dead flowers. Examination showed some webbing and caterpillar frass in most parts of the inflorescence, and caterpillars with a dark head and dark purplish body were associated with this. Two cocoons that could have been *Batrachedra* sp. were found, but nothing emerged from these and nothing emerged from the emergence box, so it cannot be established whether or not this species is a suitable food plant for a *Batrachedra* sp. or any of the Lepidoptera found in this survey, but certainly it is host to at least 1 species of Lepidoptera.

An old inflorescence of *L. spinosa* from which the male flowers had been dropped showed no sign of Lepidoptera feeding, webbing of male flowers, or cocoons.

There was light webbing and frass among dropped male flowers in an inflorescence of *Pritchardia pacifica*, but no caterpillars or cocoons matching those of *Batrachedra* spp. were found, and 19 adults of 2 other species of moths were obtained by emergence box.

The male flowers of *Ptychosperma macarthurii* are not synchronised, hence there were small numbers of scattered male flowers on the inflorescences included in the survey at both Curepe and the Royal Botanic Gardens. These and some dropped flowers caught up between the inflorescence branches at the Royal Botanic Gardens were sampled. There was no trace of webbing or frass on fresh or dropped male flowers, and no Lepidoptera were obtained by emergence box.

In the *S. romanzoffiana* inflorescence sampled, the male flowers had already mostly dropped. The inflorescence seemed completely healthy, except that the ends of many apical secondary branches were caught up in the apex of the spathe together with many male flowers, which had started to go moldy. This seemed ideal for attack by Lepidoptera, but there was no trace of caterpillar damage. A small collection of dropped male flowers was also made from the base of the palm and set up separately. No Lepidoptera emerged from either sample.

## Discussion

A preliminary spot survey of the incidence of Lepidoptera in the flowers of coconuts and selected other palms in Trinidad was carried out. The results have enabled confirmation of the presence of *B. nuciferae* to be widespread in Trinidad. Information has been generated on the feeding damage, phenology, and natural enemies of *B. nuciferae*. Coconut has been confirmed as a food plant, palmiste is a new food plant record, and several other palms have been provisionally shown to be unsuitable food plants. This information was used to assess the threat that *B. nuciferae* presents to coconut and other palms and the need for management of the moth.

### Host range of *B. nuciferae*

The results show that both coconut and *R. oleracea* were suitable hosts for *B. nuciferae* in Trinidad. No evidence was found of *Batrachedra* spp. attacking *B. major*, *B. simplicifrons*, *A. maripa*, *M. flexuosa*, *E. precatoria*, or 4 introduced palms. *Sabal mauritiiformis* was attacked by another *Batrachedra* sp., but not by *B. nuciferae*. However, while proof of attack was easily demonstrated, proof of non-attack cannot be demonstrated without more samples taken at a suitable stage. In the specific case of *B. nuciferae*, these samples should be taken in places where *B. nuciferae* is known to be present on other nearby hosts.

Because Bondar (1940a, b) recorded *B. nuciferae* feeding abundantly in the inflorescences of *Attalea* spp. in Brazil, the indigenous *A. maripa* were expected to be suitable hosts in Trinidad. However, in spite of the large amount of old damage on the old male inflorescence, this did not appear to be the case. There are several possible explanations: (1) Bondar saw similar damage due to other Lepidoptera and assumed it was due to *B. nuciferae* without rearing out specimens (he appears to have sent only specimens from coconut for identification); (2) the species of *Attalea* that Bondar observed are suitable food plants, but *A. maripa* is not (it is sometimes placed in a separate genus to other *Attalea* spp.); (3) further samples may show that *A. maripa* in Trinidad is a suitable food plant for *B. nuciferae*. The last would be the easiest to test, especially if fresh flowering male inflorescences could be sampled. It can be concluded provisionally that *A. maripa* is not a suitable food plant for *B. nuciferae*. The male flowers of *A. maripa* consist of little more than a bunch of pollen-bearing stamens (Henderson et al. 1995), so they are very different in structure from all the other palms that were sampled, and this may be a factor in the apparent unsuitability of *A. maripa* as a host.

The genus *Syagrus* is considered to be the closest relative of *Cocos* (Henderson et al. 1995), so *S. romanzoffiana*, the queen palm, is a likely host for *B. nuciferae*, not least because Bondar (1940a, b) recorded *B. nuciferae* from the inflorescence of other *Syagrus* spp. Hence, it would seem premature to conclude that *B. nuciferae* does not attack *S. romanzoffiana*, especially because this survey did not confirm that *B. nuciferae* is present in the Royal Botanic Gardens where the *S. romanzoffiana* was sampled.

In addition to the suggestion above that structural differences in flowers of *A. maripa* could be a factor in host-specificity, it seems likely that palms with synchronised flowering in an inflorescence, which then drop all male flowers almost immediately, would not be suitable hosts for *Batrachedra* spp. specialised on male pollen-producing flowers. On this basis, the observations of Essig (1971) and Ervig (1993), which indicate this pattern of flowering in *Bactris* spp. and *M. flexuosa*, mean these palms are unlikely to be suitable hosts, which was the provisional conclusion in this study.

These observations on host range should also be considered in relation to the classification of Arecaceae (Dransfield et al. 2008). *Attalea* is the only genus of indigenous palm in the same subtribe as *Cocos* (Attaleinae), and there was no evidence from this survey that *B. nuciferae* attacks *A. maripa*. Note however, that contrary to the observations reported here, Bondar (1940a, 1940b) found *Attalea* spp. to be hosts, albeit not confirmed with identified specimens. There are 17 species of indigenous palms in four subtribes which are in the same tribe as *Cocos* (Cocoseae), but the ones sampled, *Bactris* spp. (Bactridinae) and *Euterpe precatoria* (Euterpeae), were not attacked by *B. nuciferae*. However, *R. oleracea*, which is in a different tribe (Roystoneae) of the same subfamily as *Cocos* (Arecoideae), was attacked. It seems likely that because of the great diversity of pollination systems in Arecaceae (Henderson 1986) linked to a comparable diversity of inflorescence structure and phenology, specific details such as flower structure, the duration of male flowering, and the time of retention of male flowers may be more important in defining a palm's suitability as a host than strict phylogenetic relatedness.

### Impact on nut production

The observations reported here support the view that the caterpillars of *B. nuciferae* feed only on male pollen, which is only briefly available, whereas the caterpillars of other Lepidoptera species feed on other parts of the inflorescence and/or the decaying inflorescence. Careful examination of the palm inflorescences where *B. nuciferae* caterpillars were recognised showed no evidence that *B. nuciferae* caterpillars bore into male flowers or attack female flowers. This matches the observations of Bondar (1940a, b), Sánchez Soto (2004), and Sánchez-Soto and Nakano (2008). However, the possibility that more direct damage to flowers could happen at higher densities of caterpillars should not be ignored. Thus, samples of heavily invested coconut and other palm inflorescence should be carefully examined before coming to a firm conclusion about this. Nevertheless, at the population levels found, the caterpillars of *B. nuciferae* feed primarily, if not exclusively, on pollen in male flowers and do not directly damage female flowers.

Studies on coconut inflorescence damage, nutfall, and loss of yield (Taylor 1930; Corbett 1931; Cock et al 1987; Waterhouse and Norris 1987) have shown this is a complex subject with conflicting results. However, most studies suggest that the loss of male flowers is unlikely to be significant, as shortfalls in male flowers would not normally be limiting for pollination. On the other hand, damage to female flowers is implicated in loss of yield, although not conclusively. Thus, direct damage by *B. nuciferae* to male flowers would not have a direct impact on the yield and economics of coconut production or on nut production of indigenous palms.

There is also the possibility of indirect impact. For example, by tying together male flowers with silk, the microhabitat on the branches of the inflorescence and in areas where fallen flowers accumulate is changed, probably making it more suitable for both *Batrachedra* spp. and other inflorescence feeding Lepidoptera, including detritus feeders. Some of these other Lepidoptera will damage female flowers, as Bondar (1940a, b), Santana (2008), and Santana et al. (2009) reported for *Atheloca bondari* and *A. subrufella* and Bondar (1940a, b) reported for *Cadra cautella*. In this situation, the presence of *B. nuciferae* could lead to damage to female flowers, although the extent and frequency to which this happens would need to be investigated.

### Effectiveness of methods

Obtaining samples was a significant challenge due to limited availability of flowers at the selected sites in the week of the survey, and those palms which were flowering were often too tall to sample safely. The samples obtained were not necessarily at the best stage to look for flower feeding caterpillars, especially those that feed on pollen as *Batrachedra* spp. do. Nevertheless, because Lepidoptera feeding leaves recognisable traces, such as webbing, frass, and characteristic cocoons, old inflorescences where the dead male flowers were still present provided useful information.

Examination of inflorescences in the laboratory gave fairly reliable insight into some of the species present and their damage, but overlooked eggs or very young individuals and risked overlooking very low-density populations. No objective assessment of the effectiveness of the emergence boxes was made.

As a measure of population density, taking a sample at a single time point as done here unavoidably makes no allowance for eggs that would have been laid in the inflorescence after collection, nor for moths that had already emerged before the sample was taken. By making a careful examination of the material before setting it up in the emergence box, there is a qualitative control for the latter aspect, but the former could not be controlled. In some cases, *Batrachedra* moths did not start to emerge until well after the set-up of the emergence box, and it may well be that if these inflorescences had been sampled a week or two later, they would have produced more moths. Nevertheless, there was wide variation in the numbers of moths obtained from the emergence boxes, and large numbers of moths were obtained in several cases, so this method does provide a time-efficient and costeffective way to recognise the relative density of populations of Lepidoptera in the different inflorescences at the time of sampling. However, this approach could be improved by (1) standardising the stage of development of inflorescence sampled, i.e., to pollen producing; (2) separating pollen producing flowers from old dropped flowers and holding each separately for moth emergence; (3) establishing that *B. nuciferae* is present in a local area before sampling other nearby palm species to assess host specificity.

### Is *Bactrachedra nuciferae* indigenous or alien in Trinidad?

*Batrachedra nuciferae* was first found in Trinidad in 2006. However, there are two possible interpretations of this: either it is an indigenous species that had not previously been noticed and identified, or it is an alien species introduced some time before 2006. There is not definitive evidence to address this issue, but there are several points that should be considered.

The available lists of moths from Trinidad (Kaye and Lamont 1927; Lamont and Callan 1950) do not mention *B. nuciferae*. However, the lists include very few species of the socalled Microlepidoptera families (which include Batrachedridae), and those mentioned are mostly important pests recorded through studies by the Department of Agriculture (Trinidad and Tobago) entomologists and staff or students of the former Imperial College of Tropical Agriculture (St. Augustine, Trinidad). *Batrachedra nuciferae* is a small, undistinguished moth, most unlikely to be found by casual observation and collecting. It would have to be located through a study or survey of coconut pests that examined the insects associated with the inflorescence. Similarly, although at least 12 other species of Lepidoptera were reared in this study from coconut or other palm inflorescences, only 1 had previously been recorded from Trinidad. There is no evidence that any entomologists have studied what insects affect coconut inflorescence in Trinidad prior to 2006.

It is only relatively recently that the inflorescence of coconut has become easily accessible in Trinidad, thanks to the planting of dwarf hybrid varieties. When most flowering palms were too tall for casual observation, there would have been little incentive to examine the inflorescence for insects. Even in an area well-endowed with economic entomologists such as Florida, it was only in 1982 that it became known that *A. subrufella* was a damaging indigenous moth pest of coconut inflorescence (Habeck and Nickersen 1982), demonstrating how easily small Lepidoptera feeding on coconut inflorescence may be overlooked.

If *B. nuciferae* were an introduced species, it would be more likely to have no associated specialist natural enemies, whereas if it were indigenous it would be more likely to have them. This survey showed that *B. nuciferae* is parasitized by an *Apanteles* (*sensu lato*) sp. on coconut. *Apanteles* (*sensu lato*) spp. are usually specialist parasitoids, so this evidence supports an indigenous origin rather than an exotic one. Finally, if *B. nuciferae* were recently introduced in Trinidad, its distribution might be restricted, whereas it seems to occur throughout the island (F. Hosein, personal communication).

Thus, the balance of evidence does suggest that *B. nuciferae* is more likely to be an indigenous species in Trinidad than an alien species. However, none of these points are conclusive. To resolve this issue, a study on the molecular genetics of *B. nuciferae* in Trinidad (and elsewhere) would be needed. If *B. nuciferae* is a recently introduced species in Trinidad, it should have a much-reduced genetic diversity there compared to areas where it is indigenous.

### The need for control of *Bactrachedra nuciferae*

At the moment, the evidence available does not suggest that control of *B. nuciferae* should be a priority compared to other adverse factors affecting coconut and other palms in Trinidad, e.g., red palm mite (*Raoiella indica*), red ring disease (caused by the nematode *Bursaphelenchus cocophilus*), and other diseases known in the region. The limited observations in this study did not suggest that *B. nuciferae* causes direct damage or occurred at high enough densities to do so. The observations also showed that the caterpillars are attacked by an *Apanteles* (*sensu lato*) sp. parasitoid, which could be quite common. Ants were observed carrying away *B. nuciferae* caterpillars in the laboratory. Other natural enemies may well attack the eggs or pupae, but would not have been detected in this survey. Until demonstrated otherwise, it is anticipated that the action of natural enemies (predators, parasitoids, and diseases) would increase when the density of *B. nuciferae* increased, bringing any incipient outbreak under control. In extreme cases, for example if damage is observed early in the cropping cycle of newly planted dwarf varieties of coconut, further observations should be made, but as long as the inflorescences are being visited by pollinators, action to control flower pests such as *Batrachedra* spp. that do not attack the female flowers are not likely to be useful.
